# Comparing the Risk of SARS-CoV-2 Immune Resistance Evolving Across Regions in the Americas with Differing Approaches to Public Health

**DOI:** 10.3390/pathogens15070682

**Published:** 2026-06-26

**Authors:** Kenichi W. Okamoto, Luis F. Chaves, Luke Bergmann, Rodrick D. Wallace, Robert G. Wallace

**Affiliations:** 1 Department of Biology, University of St. Thomas, St. Paul, MN 55105, USA; 2 Department of Environmental and Occupational Health, School of Public Health and Department of Geography, Indiana University, Bloomington, IN 47405, USA; lfchavs@gmail.com; 3 Instituto Conmemorativo Gorgas de Estudios de la Salud, Ciudad de Panama 0816-02593, Panama; 4 Department of Geography, University of British Columbia, Vancouver, BC V6T 1Z2, Canada; luke.bergmann@gmail.com; 5 Department of Epidemiology, New York State Psychiatric Institute, New York, NY 10032, USA; 6 Agroecology and Rural Economics Research Corps, St. Paul, MN 55105, USA; rwallace24@gmail.com

**Keywords:** virus–vaccine evolution, social–evolutionary feedbacks, healthcare and wellbeing systems, Latin America

## Abstract

Public health policy foundationally impacts how pathogens spread, yet despite multiple pathogens of broader societal concern emerging, little research has examined how policy affects pathogen evolution. To evaluate this connection, we examine how varying public health approaches impact how viral immune susceptibility, including resistance to vaccines, evolves. Integrating evolutionary epidemiological modeling and critical geography, we compare how distinct public health responses early in the COVID-19 pandemic affected the potential evolution of immune evasion in SARS-CoV-2 across four territories: Costa Rica, Panama, Texas, and Uruguay. We use parameter estimates inferred from confirmed case and vaccination time series via stochastic ensemble Kalman filtering in each territory. Our analyses suggest viral immune resistance was most likely to emerge in Texas, which relied almost exclusively on vaccines for disease control. In contrast, regions with comparatively fewer health disparities that also rigorously applied interventions, such as shelter-in-place orders and household support, may have better prevented vaccine resistance from evolving. These comparative analyses highlight the key role policy choices play, potentially representing different governance goals for population health and wellbeing. We argue that such choices impact not only disease spread but also pathogen evolution along epidemiologically critical dimensions like viral immune susceptibility. Our study thus demonstrates how public health priorities drive social–evolutionary feedbacks.

## 1. Introduction

Considerable attention has been paid to how distinct public health policies impact the spread of viral pathogens during a pandemic [1,2,3,4,5,6]. Mask mandates, shelter-in-place orders, and broader interventions including healthcare access and eviction moratoriums mitigate transmission, albeit to varying degrees [7,8,9,10]. Despite the striking emergence of multiple variants of concern during the COVID-19 and other pandemics, less attention has been directed to the effects of policy upon viral evolution during pandemics. Increasingly, modelers have begun examining policy’s influence on the evolution of viral resistance to vaccines [11,12,13,14,15,16] and prevailing host immunity profiles (e.g., [17,18,19]). Such public health policies, in turn, presumably reflect a polity’s health and wellbeing goals (e.g., [11]). Most modeling studies that have explored the emergence of immune evasion in SARS-CoV-2 have focused their analyses on a single region (e.g., [12,15,16,17,19]) or used models less tethered to data that enabled them to explore wider regions of parameter space (e.g., [11,20]). A notable exception is the work of [13], but the focus there was on motivating the analysis using different observed rates of vaccine uptake across reasons while simulating the effects of hypothetical social-distancing measures rather than a comparative analysis of how varying approaches to public health policies across societies affects the evolution of vaccine resistance. Thus, by comparing the prospects for viral evolution in locales adopting different public health policies during pandemics, we can highlight how broader societal choices might drive the trajectory of pathogen evolution along epidemiologically relevant dimensions.

Here, we take the COVID-19 pandemic as a case study and analyze mathematical models linking pandemic outcomes to viral evolution using parameters estimated with time-series data of COVID-19 cases and vaccination records from four regions in the Americas: Costa Rica, Panama, Texas, and Uruguay. As we argue below, these regions contain several similarities while also differing markedly in their health policy choices as sufficiently coherent units early in the COVID-19 pandemic. We comparatively evaluate whether distinct public health responses since the start of the pandemic have set the trajectory for the evolution of immune evasion in SARS-CoV-2 across different regions. In contrast to previous studies modeling the evolution of vaccine resistance in SARS-CoV-2, our strategy enables us to interpret the results of our evolutionary epidemiological modeling through the lens of critical geography. In particular, based on these data and the wider theoretical framework we propose, we evaluate a growing literature suggesting that vaccine resistance is likely to emerge in locales relying almost exclusively on vaccines for disease control (e.g., [17,20,21]). In contrast, we find that regions that also rigorously applied nonpharmaceutical public health interventions (e.g., shelter-in-place orders and household support) were expected to better prevent vaccine resistance from evolving. Our study underscores the necessity of testing the impacts of policy choices beyond pharmaceutical adaptation on pandemic outcomes, not only with respect to disease spread concerning pathogen evolution.

## 2. Model Development

### 2.1. The Baseline Epidemiological Model

We begin our analysis of the risk of immune evasion evolving by considering a host population in which an ancestral, immune-susceptible strain (A) circulates. Although our model is developed and parameterized for SARS-CoV-2 during the early years of the COVID-19 pandemic, we suspect our basic approach will apply to most viruses that exhibit: (i) frequency-dependent transmission (e.g., [22,23]), (ii) limited effects of virulence-induced host mortality on epidemiological dynamics (e.g., [24,25,26,27,28]), and (iii) a host that can mount an adaptive immune response following infection that lasts for the duration of the time period over which an immune-evading strain can evolve.

In the absence of vaccination or epidemiologically relevant variability in the susceptibility profiles of human or viral populations, the ancestral viral strain is assumed to circulate in the host population following an S → E → I → R (Susceptible–Exposed–Infectious–Recovered) compartmental model over the time horizon that we investigate. The ancestral strain spreads through frequency-dependent transmission with a per capita force of infection equal to $\beta_{A}I_{A}\left(t\right)/N$, where N is the total host population density, $I_{A}\left(t\right)$ is the density of hosts infectious with the ancestral strain, and $\beta_{A}\left(t\right)$ describes the ancestral strain’s time-varying infection coefficient. For the same viral strain, variation in this infection coefficient depends, in turn, on prevailing societal responses to viral spread. The total host population density is determined by the density of hosts susceptible to infection by the ancestral strain ($S_{A}\left(t\right)$), the density of hosts exposed to the ancestral strain ($E_{A}\left(t\right)$), and the density of hosts who have recovered from infection with the ancestral strain ($R_{A}\left(t\right)$) (i.e., in the absence of strain diversity or vaccination, $N\left(t\right)=S_{A}\left(t\right)+I_{A}\left(t\right)+E_{A}\left(t\right)+R_{A}\left(t\right)$).

These considerations suggest the following system of differential equations as a baseline in the absence of vaccination and viral evolution (i.e., when only a single, ancestral viral strain (A) is present):

(1) $$\begin{array}{l} S_{A}^{\prime }\left(t\right)\;=\;-\beta_{A}\left(t\right)I_{A}\left(t\right)\frac{S_{A}\left(t\right)}{N}\\ E_{A}^{\prime }\left(t\right)\;=\;\beta_{A}\left(t\right)I_{A}\left(t\right)\frac{S_{A}\left(t\right)}{N}-\sigma E_{A}\left(t\right)-qE_{A}\left(t\right)\\ I_{A}^{\prime }\left(t\right)\;=\;\sigma E_{A}\left(t\right)-\gamma I_{A}\left(t\right)-qI_{A}\left(t\right)\\ R_{A}^{\prime }\left(t\right)\;=\;\gamma I_{A}\left(t\right). \end{array}$$

Here, σ and γ represent the incubation period and recovery rate, respectively. The effect of non-pharmaceutical interventions (e.g., isolation) depends on the strength (q) of the effect of removing exposed and infectious hosts from the population.

For purposes of the present analyses, we follow, (e.g., [13,27,28,29]) and consider disease-induced mortality to not substantively alter the pathogen’s dynamics, in addition to assuming that the total host population density is approximately constant over the time scale of concern. In effect, the latter assumption implies that the magnitudes of births, deaths, immigration, and emigration effectively cancel each other out over the time horizon we analyzed.

We then relax the assumption of immunity being acquired solely via infection by allowing hosts susceptible to infection by the ancestral, vaccine-susceptible strain to become vaccinated at a time-varying per capita rate (v(t)). Thus, our S → E → I → R model is modified so that vaccinated hosts are diverted into a compartment (V) representing fully vaccinated hosts, and the dynamics of hosts susceptible to the ancestral strain become $S_{A}^{\prime }\left(t\right)=-\beta_{A}\left(t\right)I_{A}\left(t\right)\frac{S_{A}\left(t\right)}{N}-v\left(t\right)S_{A}\left(t\right)$, while the density of vaccinated hosts changes according to $V_{A}^{\prime }\left(t\right)=v\left(t\right)S_{A}\left(t\right)$. We assume fully vaccinated hosts are unable to transmit the ancestral strain but still circulate in the host population. Thus, after vaccinations begin, $N=S+I+E+R_{A}+V$.

### 2.2. Modeling the Evolution of Immune Evasion

**Figure 1:**
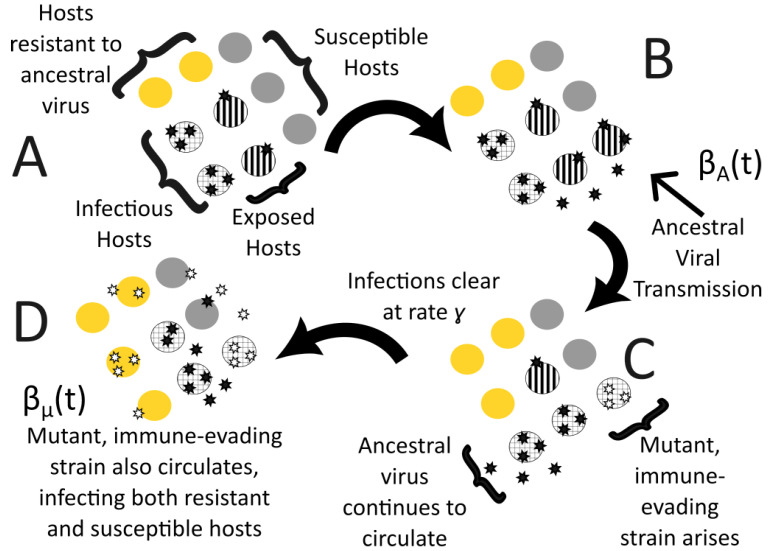
The evolutionary epidemiological model for the emergence of a novel, immune-evading viral strain. (**A**,**B**) The immune-susceptible strain (*A*, black stars) can only infect fully susceptible hosts (gray circles), rendering them exposed (black, striped circles). Viral strain *A* cannot infect vaccinated and previously infected hosts (yellow circles). (**C**) An immune-evading variant (*µ*, white stars) is modeled to evolve to replace virus *A* first inside a single host. (**D**) Whether *µ* subsequently spreads to new hosts depends on prevailing patterns of immunity and the availability of susceptible, vaccinated, and recovered hosts.

Within a single infectious host, a mutation (or series of mutations) that produces an immune-resistant variant that replaces the immune-susceptible strain is modeled to arise in that single host. The immune-resistant variant subsequently infects others hosts. Figure 1 summarizes the underlying evolutionary epidemiological dynamics. We therefore modify model (1) to assess the evolutionary invasibility of an immunity-evading strain (μ) of the virus in the host population in which only the ancestral virus strain circulates, i.e., until the host in which the mutant arose infects other hosts with the novel strain, all other infectious hosts continue to be infected by the ancestral strain (A).

We consider a situation where there are $S_{u}$ hosts that have never been infected by either strain nor vaccinated, as well as $S_{v}$ (i.e., $R_{A}(t)$ + V(t)) hosts that have either been vaccinated or previously infected by—and are now immune to—strain A. We further neglect coinfection, and immunity to strain μ is assumed to confer immunity to strain A. We allow for the possibility that host susceptibility to the novel strain, as well as how quickly exposed hosts become fully infectious (i.e., their incubation period), potentially differs among hosts who have and have never been infected with or vaccinated against the ancestral strain. However, upon becoming infectious with the novel strain, the host’s prior infection history is modeled to not impact their potential to infect other hosts or the viral clearance rate. These assumptions can be reasonable if prior infection by the ancestral strain or vaccination confers, for example, some degree of cross-over immunity. Such effects could potentially prevent or delay a subset of exposed hosts from becoming infectious, but, in cases where they do eventually become infectious with the novel strain, hosts vaccinated or previously infected with the ancestral strain can potentially exhibit a viremia comparable to that of hosts who have never been immunized or infected. Thus, the evolutionary epidemiological model can be described by the following system of equations when the new, immune-evading strain emerges:

(2) $$\begin{array}{l} S_{u}^{\prime }\left(t\right)\;=\;-\beta_{A}\left(t\right)I_{A}\left(t\right)\frac{S_{u}\left(t\right)}{N}-v\left(t\right)S_{u}\left(t\right)-\beta_{\mu }I_{\mu }\left(t\right)\frac{S_{u}\left(t\right)}{N}\\ E_{A}^{\prime }\left(t\right)\;=\;\beta_{A}\left(t\right)I_{A}\left(t\right)\frac{S_{u}\left(t\right)}{N}-\sigma E_{A}\left(t\right)-qE_{A}\left(t\right)\\ I_{A}^{\prime }\left(t\right)\;=\;\sigma E_{A}\left(t\right)-\gamma I_{A}\left(t\right)-qI_{A}\left(t\right)\\ S_{v}^{\prime }\left(t\right)\;=\;\gamma I_{A}\left(t\right)+v\left(t\right)S_{u}\left(t\right)-\beta_{v}I_{\mu }\left(t\right)\frac{S_{v}\left(t\right)}{N}\\ E_{\mu }^{\prime }(t)\;=\;\beta_{\mu }I_{\mu }\left(t\right)\frac{S_{u}\left(t\right)}{N}-\sigma_{\mu }E_{\mu }(t)-qE_{\mathrm{v, \mu}}\left(t\right)\\ E_{v,\mu }^{\prime }(t)\;=\;\beta_{v}I_{\mu }\left(t\right)\frac{S_{v}\left(t\right)}{N}-\sigma_{v,\mu }E_{v,\mu }\left(t\right)-qE_{v,\mu }\left(t\right)\\ I_{\mu }^{\prime }\left(t\right)\;=\;\sigma_{v,\mu }E_{v,\mu }\left(t\right)+\gamma I_{\mu }\left(t\right)+qI_{\mu }\left(t\right)\\ R_{\mu }^{\prime }\left(t\right)\;=\;\gamma I_{\mu }\left(t\right), \end{array}$$

where the γ,σ,N, and $\beta_{A}(t)$ parameters are as above. $E_{\mu }(t)$ and $E_{v,\mu }\left(t\right)$ are the densities of hosts exposed to the mutant pathogen who have never and have, respectively, been vaccinated or infected by the ancestral strain, while $I_{\mu }$(t) is the density of hosts infectious with strain μ and $R_{\mu }\left(t\right)$ is the density of hosts that have recovered (and are therefore immune to) the immune-evading strain. $\sigma_{\mu }$ and $\beta_{\mu }$ are the incubation period and infectivity, respectively, of the immune-evasive strain (μ), viz., hosts that have never been infected, and $\sigma_{v,\mu }$ and $\beta_{v}$ are corresponding quantities for hosts that have already been infected by the ancestral strain. In contrast to $\beta_{A}\left(t\right),$ $\beta_{\mu }$ and $\beta_{v}$ are modeled as constants, as the mutant strain is rarer and, thus, the magnitude of its infection coefficient can reflect lower levels of heterogeneity (e.g., due to transmission among initially less diverse host contact networkers, lower within-strain genetic variation, more uniformity in free-living virion counts due to the limited number of infected hosts, et.), at least very early in the emergence of an immune-evading strain. Furthermore, because comparisons of the infectious period suggest consistency across strains (e.g., [30]), we do not distinguish between the ancestral and mutant strains’ recovery rates (γ). To contrast the different evolutionary effects of public health interventions, we model a situation where hosts infected by SARS-CoV-2 are potentially removed from the host population at a rate of q. We assume that this rate (q) is reflective, in part, of the testing intensities and willingness and ability of residents to comply with isolation and quarantining recommendations across different polities.

Under these conditions, the average quantity of secondary infections ($\begin{array}{c} R_{\mu } \end{array}$) with mutant strain μ characterizes the evolutionary invasibility of the novel strain. The average quantity of secondary infections ($\begin{array}{c} R_{\mu } \end{array}$) can be derived from the evolutionary epidemiological model as follows. First, we consider a host population in which only the ancestral viral strain (A) circulates. If a mutant strain (μ) emerges in a single host, it will be able to initially spread in the host population if, on average, the mutant strain manages to infect more than one further host.

### 2.3. Determining the Average Number of Secondary Infections for the Mutant Strain

To determine how many secondary infections the mutant strain (μ) causes, we begin by analyzing the behavior of the evolutionary epidemiological model when a mutant strain is initially very rare.

Our measure ($\begin{array}{c} R_{\mu } \end{array}$), i.e., the average number of secondary infections for the mutant strain, is a time-varying quantity during the study period we examined. To determine its expression, we use a modified version of the basic reproductive number derived using the next-generation operator (e.g., [31,32,33], but see [34]).

Following [31,32], the “positive matrix” (***F***) for the infected compartments (i.e., hosts exposed to the mutant virus that had and had not been previously vaccinated, as well as hosts infectious with the mutant virus) of Equation (2) is given by ***F***= $\left[\begin{array}{ccc} 0 & 0 & \frac{S_{U}(t)\;\beta_{\mu }}{N}\\ 0 & 0 & \frac{S_{v}{(t)\beta }_{v}}{N}\\ \sigma_{\mu } & \sigma_{v,\mu } & 0 \end{array}\right]$, and the “outflow matrix” (***d***) for Equation (2) is given by ***d*** = $\left[\begin{array}{ccc} q+\sigma_{\mu } & 0 & 0\\ 0 & q+\sigma_{v,\mu } & 0\\ 0 & 0 & q+\gamma \end{array}\right],$ whose inverse ($d^{-1}$) is given by $\left[\begin{array}{ccc} \frac{1}{q+\sigma_{\mu }} & 0 & 0\\ 0 & \frac{1}{q+\sigma_{v,\mu }} & 0\\ 0 & 0 & \frac{1}{q+\gamma } \end{array}\right]$.

We note that the difference between these two matrices allows us to characterize the local linearization of the mutant, immune-evading strain’s dynamics when infections by that strain are initially rare in Equation (2). This can be seen by noting that ***F*-*d*** = $\left[\begin{array}{ccc} -q-\sigma_{\mu } & 0 & \frac{S_{U}{(t)\beta }_{\mu }}{N}\\ 0 & -q-\sigma_{v,\mu } & \frac{S_{v}(t)\beta_{v}}{N}\\ \sigma_{\mu } & \sigma_{v,\mu } & -q-\gamma \end{array}\right]$, and multiplying this matrix by a vector of the relevant state variables $\left[\begin{array}{c} E_{\mu }(t)\\ E_{v,\mu }(t)\\ I_{\mu }(t) \end{array}\right]$ recovers the right-hand sides of the expressions in Equation (2).

Thus, the next-generation operator ([31,32]) can be determined as the dominant eigenvalue of the product matrix expressed as ***F***$\cdot \;d^{-1}$, i.e., $\left[\begin{array}{ccc} 0 & 0 & \frac{S_{U}{(t)\beta }_{\mu }}{N\left(q+\gamma \right)}\\ 0 & 0 & \frac{S_{v}{(t)\beta }_{v}}{N\left(q+\gamma \right)}\\ \frac{\sigma_{\mu }}{\left(q+\sigma_{\mu }\right)} & \frac{\sigma_{v,\mu }}{\left(q+\sigma_{v,\mu }\right)} & 0 \end{array}\right]$. Because all quantities in the matrix are non-negative, the spectral radius of ***F***$\cdot \;d^{-1}$ is given by its dominant eigenvalue.

The resulting eigenvalues of ***F***$\cdot \;d^{-1}$ are 0 and $\pm \frac{\sqrt{\frac{S_{U}{(t)\beta }_{\mu }\;}{N}\sigma_{\mu }(q+\sigma_{v,\mu })+\frac{S_{v}{(t)\beta }_{v}}{N}(q{+\sigma_{\mu })\sigma }_{v,\mu }}}{\sqrt{\left(\gamma +q\right)\left(q{+\sigma }_{\mu })(q+\sigma_{v,\mu }\right)}}$; as all terms under the square root are non-negative, the dominant eigenvalue (spectral radius) of ***F***$\cdot \;d^{-1}$ is

(3) $$R_{\mu }=\frac{\sqrt{\frac{S_{U}{(t)\beta }_{\mu }}{N}\sigma_{\mu }(q+\sigma_{v,\mu })+\frac{S_{v}{(t)\beta }_{v}}{N}(q{+\sigma_{\mu })\sigma }_{v,\mu }}}{\sqrt{\left(\gamma +q\right)\left(q{+\sigma }_{\mu })(q+\sigma_{v,\mu }\right)}},$$

which constitutes the time-varying evolutionary invasibility ($\begin{array}{c} R_{\mu } \end{array}$) of the mutant viral strain (μ).

We highlight how the evolutionary invasibility ($\begin{array}{c} R_{\mu } \end{array}$) of strain μ depends not only on the mutant and ancestral strains’ phenotypes but also on the prevailing commitment to effective public health surveillance across the polities we examined. This is because such commitments alter the composition of host populations involved in the infection cycle, as well as several key parameters including the infection coefficient and, hence, the ecological selective forces operating on the pathogen strain.

## 3. Model Analyses and Results

### 3.1. Study Regions for Comparative Analysis

To assess how distinct public health responses could drive pathogen evolution, we compared the risk of vaccine resistance emerging in four territories in the Americas: Costa Rica, Panama, Texas, and Uruguay. We chose these polities for our comparative analysis because despite their comparable baseline socioeconomic and population health profiles, they exhibited distinct public health responses early in the SARS-CoV-2 pandemic.

At first glance, the four territories share much in common. All four territories have relatively high Human Development Indices (HDIs) for the Western Hemisphere, powerful government institutions, and very large urban and periurban populations concentrated around a few metropolitan areas that grew rapidly from the mid-twentieth century on ([35,36]). Three of the territories (Costa Rica, Panama, and Texas) express comparable Gini ratios (0.47–0.51; [37,38]) similar to levels found in Uruguay until recently (whose Gini index has declined from about 0.45 in 2009 to 0.41 in 2021 [37]). Additionally, all four territories exhibit substantial contrasts in health outcomes among residents along geographic, ethnic, and socioeconomic gradients ([39,40]). Nevertheless, health-outcome inequities in domains such as coverage and access to preventative care are, in general, less pronounced in Costa Rica and Uruguay than in Texas and Panama ([35,41,42,43,44]).

Despite these similarities, the four territories we compare differed substantively in the public health policies adopted in response to the early COVID-19 pandemic. In particular, the University of Oxford’s COVID-19 Government Response Tracker, which combines pharmaceutical and nonpharmaceutical interventions to characterize public health policies through 2022 ([45]), shows Costa Rica, Panama, and Uruguay engaged in control efforts combining clinical, behavioral, and socioeconomic interventions. During the early stages of the pandemic, Uruguay, in particular, exhibited considerable success in controlling the SARS-CoV-2 outbreak ([41,46]).

In contrast, the same Oxford Index identified Texas’s pandemic responses as emblematic of COVID-19 public health policies in many parts of the U.S., with minimal time under shelter-in-place orders, near-total early reopening of businesses, and ending of mask mandates despite new waves of SARS-CoV-2 infection ([47]). Although Texas was among the first ten U.S. states to enact universal (16 years and older) access to SARS-CoV-2 vaccination, by August 2023, only 64% of Texans were fully vaccinated, a figure that dropped to 17% for the 2023–2024 dose according to CDC surveys [47,48,49]. Thus, despite its superficial similarities to other heavily urbanized, relatively high-HDI territories in the Western Hemisphere, Texas presents a very different public health policy profile than the other three locations. This allows for a sharp comparison of the epidemiological contexts out of which SARS-CoV-2 vaccine resistance could evolve.

### 3.2. Assessing the Risk of SARS-CoV-2 Immune Evasion Evolving in Each Territory

To evaluate the possible impacts of local public health policy on the evolution of vaccine resistance and immune evasion more broadly, we applied our stochastic model to quantify the risk that a mutant SARS-CoV-2 variant capable of evading immunity emerges 170 days following vaccine rollout in each of the territories described above.

Broadly, our basic strategy is described as follows. First, we estimate the daily infection coefficient ($\beta_{A}\left(t\right)$) of the ancestral strain (*A*) and the density ($S_{U}\left(t\right)$) of hosts never infected with the virus in each polity from daily (t) data on incidence, prevalence, and vaccination using an ensemble Kalman filtering regime. Next, we determine the average number ($\begin{array}{c} R_{\mu } \end{array}$) of secondary infections by single, immune-evading mutant strain μ arising from a single host in which strain A has mutated to strain μ in each territory. Because the immune-evading phenotype evolves out of the ancestral strain’s genotypic composition, which is reflective of past viral demography, and vaccination can disrupt the prevailing transmission dynamics of the ancestral strain, we model the immune-evading strain as being able to infect completely susceptible hosts (i.e., $S_{U}\left(t\right)$) at a magnitude comparable to that of the ancestral strain’s ability to infect susceptible hosts at the start ($t_{0}$) of vaccinations (i.e., $\beta_{A}\left(t_{0}\right)$). Once it appears, the immune-evading strain’s invasibility ($\begin{array}{c} R_{\mu } \end{array}$) is, in turn, modeled as a time-dependent quantity whose magnitude depends, in part but crucially, upon the density ($S_{U}\left(t\right)$) of entirely immune-naive hosts infected at time t (Section 2.3, Equation (3)). Our goal is therefore to characterize how the invasibility of the mutant strain (μ) into the host population depends on (i) the phenotypic consequences of the mutation events giving rise to μ viz. its ability to infect hosts never exposed to, as well as hosts immune to, the ancestral strain and (ii) the epidemiological profile (number of completely susceptible hosts, vaccinated hosts, etc.) of the host population when the mutant emerges.

To quantify these conditions, daily data recording total population size, as well as incidence, cumulative incidence, fatalities from SARS-CoV-2, and vaccinations against SARS-CoV-2 were collated for four polities in the Western Hemisphere (Panama, Costa Rica, Uruguay (collated as described in [35]), and Texas (https://dshs.texas.gov/coronavirus/AdditionalData.aspx, accessed on 18 August 2021), with the underlying data available at https://github.com/kewok/immune_evasion, accessed on 22 May 2026). Each dataset is concatenated to include only data following the beginning of vaccinations, and based on [50,51], we use an incubation period (which determines σ) of 8.29 days and an infectious period (which determines γ) of 5 days. Varying the incubation periods for the mutant strain over several orders of magnitude did not alter our conclusions, so we present results for when $\sigma =\sigma_{v,\mu }=\sigma_{\mu }$. *q* was determined according to [52], using testing rates as a proxy for the rate of removal of infected hosts.

For all locations, following the onset of vaccinations, the initial number of exposed individuals ($E_{0}$) is assumed to be a proportion (π) of $I_{0}$ initially infectious individuals, with π∼N(γ/σ,0.25) [27], and the initial number of vaccinated individuals ($V_{0}$) is given by the number of people fully vaccinated in the dataset or, in the case of Texas, 0. The total population size (N) for each territory is determined based on [52] and the Texas Department of Public Health. Thus, $S_{0}=N-E_{0}-I_{0}-R_{0}-V_{0}$. Once initial conditions are determined, a time-lagged per capita vaccination rate is calculated. Thus, the daily fraction (v(t)) of susceptible hosts vaccinated changes over time. Because of differences in the reported data, the routine for calculating the per capita vaccination rate is somewhat distinct for Texas relative to the other polities. We assume that it takes 30 days following the initial administration of vaccination for individuals to enter the vaccinated compartment.

These data are then used to seed an Ensemble Kalman Filter (EnKF) algorithm to determine the daily infection coefficient ($\beta_{A}\left(t\right)$) for the ancestral, immune-susceptible strain. Our approach loosely follows the strategy described by [27]. Briefly, using the initial conditions and daily vaccination rates, we use the Gillespie algorithm [53] to simulate *M* = 100 stochastic realizations of an S→E→I→R→V model with daily varying vaccination rates (v(t)) to identify distinct epidemiological trajectories up to a given date (T) for a given infection coefficient ($\beta_{A}$) ranging from 0.01 to 3.5 in increments of 1/100. This enables us to approximate the following negative log likelihood for a time point ($t_{k}$):

(4) $$L\left(t_{k},\beta_{A}\right):=\frac{1}{2}\frac{{\left|Hm_{f}\left(t_{k}\right)-y_{obs}\left(t_{k}\right)\right|}^{2}}{HP_{f}\left(t_{k}\right)H^{T}+\rho }+\frac{1}{2}log\left(HP_{f}\left(t_{k}\right)H^{T}+\rho \right)$$

(Equation (13) of [27]), where $y_{obs}\left(t_{k}\right)$ is the cumulative number of cases up to time point $t_{k}$, H = (0, 0, 1, 1), $m_{f}\left(t_{k}\right)$ is an empirical mean of the average of *M* Gillespie realizations (in $R^{4}$) of the model state ${\left(S,E,I,R\right)}^{T}$ at time $t_{k}$, and $P_{f}\left(t_{k}\right)$ is the empirical covariance matrix defined analogously with averages ($m_{f}\left(t_{k}\right)$). To retain consistency and continuity with the implementation in previous work, we follow [27] in adopting an observation model ($Y\left(t_{k}\right)$) with an additive Gaussian error term for $y_{obs}\left(t_{k}\right)$ with mean zero and variance (ρ), which, as in [27], we set to 10. An EnKF is then applied on the 100 trajectories to determine each value of $\beta_{A}\in \left[0.01,3.5\right]$ on each date for each polity for the ancestral strain by minimizing the cumulative negative log likelihood:

(5) $$L_{cum}\left(\beta_{A}\right)=\Sigma_{t_{k}=t_{min}}^{t_{max}}L\left(t_{k},\beta_{A}\right),$$

i.e., the value of $\beta_{A}$ minimizing the time-dependent log-likelihood function [27] for a particular location.

To apply the stochastic Gillespie simulation when the vaccination-rate parameter v(t) is, itself, time-varying, the underlying stochastic realization of SEIR + V is simulated by the SEIR_custom function from the ssar R package ver. 0.0.0.9000 ([54]). Briefly, the strategy for conducting a Gillespie simulation with a time-varying rate parameter calculates the propensity function (e.g., [53]) not only based on the value of the state variables but also based on the values of an independent, time-varying rate parameter (i.e., v(t)) using the implementation of the modified Next Reaction Method (e.g., [55]) from [54]. All calculations described above were performed on the Mangi cluster at the Minnesota Supercomputing Institute at the University of Minnesota, Twin Cities. All underlying code is released under the GNU Public License v3 [56] and is freely available at https://github.com/kewok/immune_evasion.

### 3.3. Comparative Risk Profiles for an Immune-Evading Strain Evolving Across Territories

**Figure 2:**
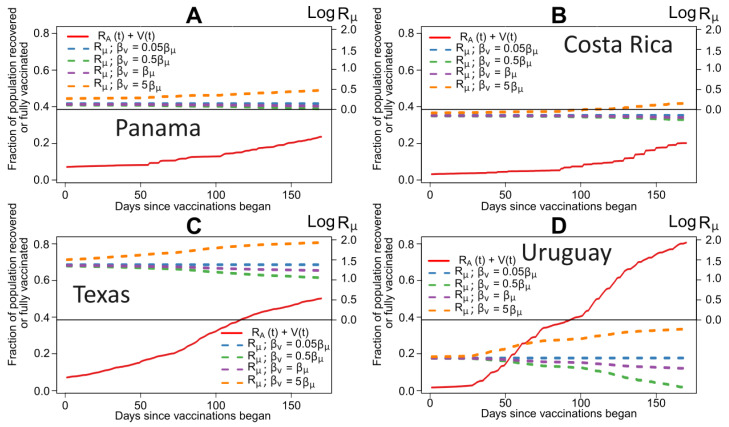
Exposure via infection or vaccination (*R_A_*(*t*) + *V*(*t*), Equation (1); solid red line) and evolutionary invasibility ($R_{\mu }$; dashed lines) of an immune-evading mutant strain (μ) across 170 days after the start of vaccinations in four Western Hemisphere territories we analyze: (**A**) Panama, (**B**) Costa Rica, (**C**) Texas, and (**D**) Uruguay. In all panels, the solid black lines represent the invasibility threshold (log $R_{\mu }$ = 0, i.e., $R_{\mu }$ = 1). We illustrate daily invasibilities of mutants capable of infecting hosts immune to the ancestral strains at 0.05–5 times their ability to infect immunologically naive hosts.

We assess how readily a novel, immune-evading strain spreads in each territory when an initially monomorphic, immune-susceptible virus is circulating. We find the risk of an immune-evading strain evolving differs qualitatively across the territories of the Western Hemisphere under consideration here. Figure 2 shows that SARS-CoV-2 exposure and immunity are steadily low in Costa Rica and Panama across the 170 days after vaccinations begin, low but slowly rising in Uruguay, and sharply rising in Texas (see also Supplementary Figure S1). To aid interpretability—in particular, to compare the responses to differences in the mutant’s potential transmissibility—the lines in Figure 2 are calculated based on the values predicted using the ensemble mean-score parameter estimates for each territory obtained from the stochastic EnKF parametrization (Supplementary Figure S2).

Our results show that as the vaccination campaign begins, an immune-evading strain is readily selected for in Texas (found above the invasibility threshold of $R_{\mu }$ = 1, log $R_{\mu }$ = 0 in Figure 2). Immune evasion can also evolve in Panama, but the mutant strain spreads considerably slower there than in Texas (Figure 2A vs. Figure 2C). In Costa Rica, only a high level of mutant transmission among hosts immune to the ancestral strain (*β_v_* = 5 *β_μ_*) permits immune evasion to evolve—and then, only very slowly well after vaccinations begin. Even with rising exposure (solid red line), any immune-resistant strain appears highly unlikely to successfully emerge in Uruguay.

These comparative results suggest that the risk of an immune-evading SARS-CoV-2 strain evolving depends on the impacts of territorial public health policies on the ancestral strain’s epidemiology at the time the mutant emerges. Texas, the territory most likely to eschew nonpharmaceutical interventions, provides an environment conducive to strong selection favoring the evolution of an immune-evading strain. Indeed, the fact that an immune-evading mutant could rapidly spread in Texas, even if it transmits two orders of magnitude slower than the ancestral strain (Figure 2C, dashed, light-blue line), highlights the strong selective pressure created by prevailing epidemiological conditions there. In contrast, among the remaining territories where nonpharmaceutical public health policies were aggressively pursued, only Panama is predicted to consistently allow for the evolutionary emergence of an immune-evading virus—and then, at a rate considerably slower than in Texas.

## 4. Discussion: Implications for Health and Well-Being

Our mathematical model, when applied to field data, suggests that the risk for the evolution of an immune-evading strain strongly depends upon daily viral transmission dynamics. To the extent that differences in public health policies explain differences in daily viral transmission dynamics, our analyses imply that the risk of a vaccine-resistant virus emerging is also shaped by public health policy.

By applying a comparative framework to four territories in the Americas with several key similarities, we argue that differences in public health policy can be a crucial variable insofar as the evolution of immune evasion is concerned.

These public health campaigns have clearly been in flux. Panama eventually converged upon a full-spectrum response: masks, vaccines, and household support. Costa Rica’s nonpharmaceutical program pivoted to depending largely on vaccines alone beginning in 2021. Uruguay’s startling early successes ([41,46]) were subsequently mitigated by demands to reopen the economy.

The case of Texas provides an especially sharp contrast to the other territories. There, SARS-CoV-2 policies were relegated primarily to local rather than regional responses, leading to considerable geographic disparities in the COVID-19 burden [57]. (The territory’s pandemic outcomes proved uneven along other axes. For instance, SARS-CoV-2 diagnoses and deaths in the Hispanic and Black populations (which, together, constitute a majority of the state’s residents) were notably elevated throughout the territory [58]). Moreover, not only was Texas one of the first regions in the United States to end mask mandates, but it also substantively lagged the rest of the United States in vaccine uptake early in the pandemic, likely leading to higher fatalities [48,59]. Thus, excepting certain primarily urban localities, Texas’s pandemic response exhibited a markedly different approach compared to the other territories we analyzed.

We need to be explicit about the scope of our analyses. Our aim here is not to prove that SARS-CoV-2 immune evasion evolved, or will evolve, in Texas in contrast to the other three territories. Rather, what we argue, following a growing literature, is that the prevailing approach to public health policy eschewing nonpharmaceutical interventions adopted in territories like Texas creates an ecological context strongly conducive to the evolution of an immune-evading SARS-CoV-2 strain. Our analyses demonstrate a stark contrast to the three other Western Hemisphere territories that embraced a spectrum of disease management strategies, including, of course, early vaccinations. In those other territories, our analyses suggest public health policies created epidemiological (i.e., selective) conditions that can more readily control the evolution of an immune-evading strain.

Thus, we argue that a program of integrated pharmaceutical and nonpharmaceutical interventions can remove the epidemiological space, that is, the selective forces, that SARS-CoV-2 needs to evolve vaccine resistance and other adaptive immune-evading changes (e.g., [11,60,61]).

More broadly, our results highlight an emerging critical theme in evolutionary epidemiology: the broader social landscapes in which pathogens evolve drive disease outbreaks [43,62]. The causes for the evolution of diseases are found as much in the field of the social determinants of health as in the object of the pathogen or patient population. In turn, the “social determinants” of health reflect *societal determinations*, as they embody how specific structural decisions imprint upon health outcomes [63,64,65]. Our comparative analysis highlights how such structural practices set many of the boundary conditions in time and space over which subsequent vaccine resistance in SARS-CoV-2 and any pandemics to follow likely evolve long before any vaccination campaign is begun.

While our study focused on the evolution of immune evasion once an epidemic is underway, the analysis contributes to our understanding of how policy choices and societal structures help drive the very emergence of novel pathogens. This research suggests that state and parastatal institutions should consider supporting programs in public health in the broadest sense; for instance, promoting healthy socioenvironmental relations as fostered by agroecology and community-controlled forestry may mitigate the evolution of zoonotic spillover [62,66]. Patients, researchers, and clinicians need to consider supplementing—and in some cases, replacing—exclusively biomedical approaches and privatized healthcare with grassroots approaches and more autonomous healthcare priorities and mobilization [67] that better retard new pathogens emerging and evolving in human populations in the first place (e.g., [66,67,68]). Such policy adjustments better place public health and economic development on mutually supportive trajectories [69].

Our results therefore suggest that pursuing proactive and structural policy changes across public health programs both locally and transnationally could not only prevent novel pathogens from emerging out of the spillover queue but could be vital in maintaining effective disease control measures against the specter of pathogen evolution, as they reflect how socioeconomic forces drive health outcomes [70]. Just as understanding teleconnections has become important in atmospheric and land-change science [71,72], global health research and practice must take account of the ways spatiotemporal dynamics of infections are determined beyond the patterns of pathogen spread and of the more-than-local social relationships found across people and places (e.g., [73,74]).

## Data Availability

The data presented in this study are openly available in Github at https://github.com/kewok/immune_evasion.

## Supplementary Materials

The following supporting information can be downloaded at: https://www.mdpi.com/article/10.3390/pathogens15070682/s1, Figure S1: Number of people fully vaccinated since the onset of vaccination for each territory. Figure S2: Estimates for susceptible hosts and the transmission coefficient for each territory.
