# Supplementary figures and images for: Comparing the Risk of SARS-CoV-2 Immune Resistance Evolving Across Regions in the Americas with Differing Approaches to Public Health
Source: Pathogens. 2026 Jun 26;15(7):682. doi: 10.3390/pathogens15070682 (PMC13414787; doi:10.3390/pathogens15070682)

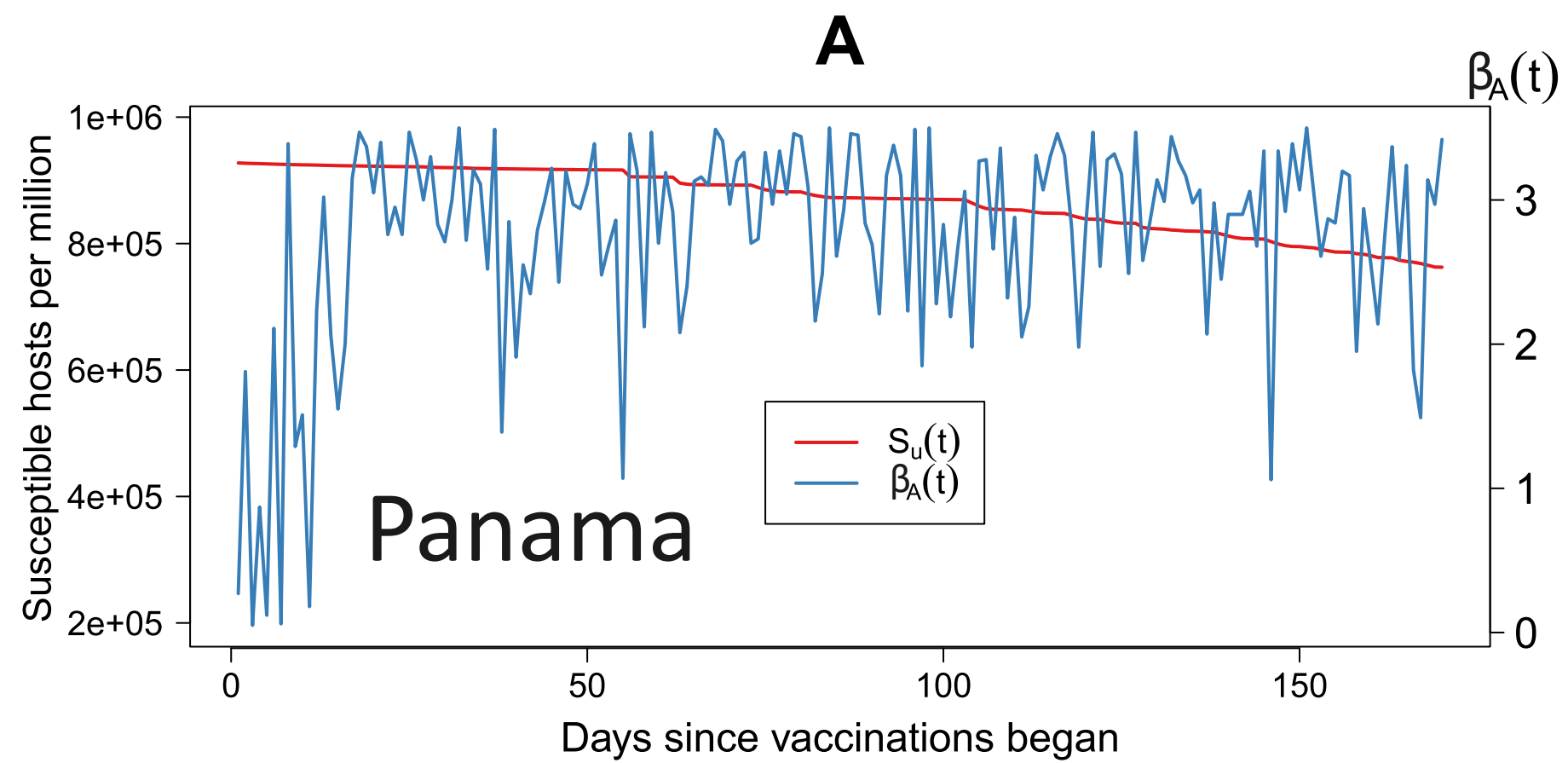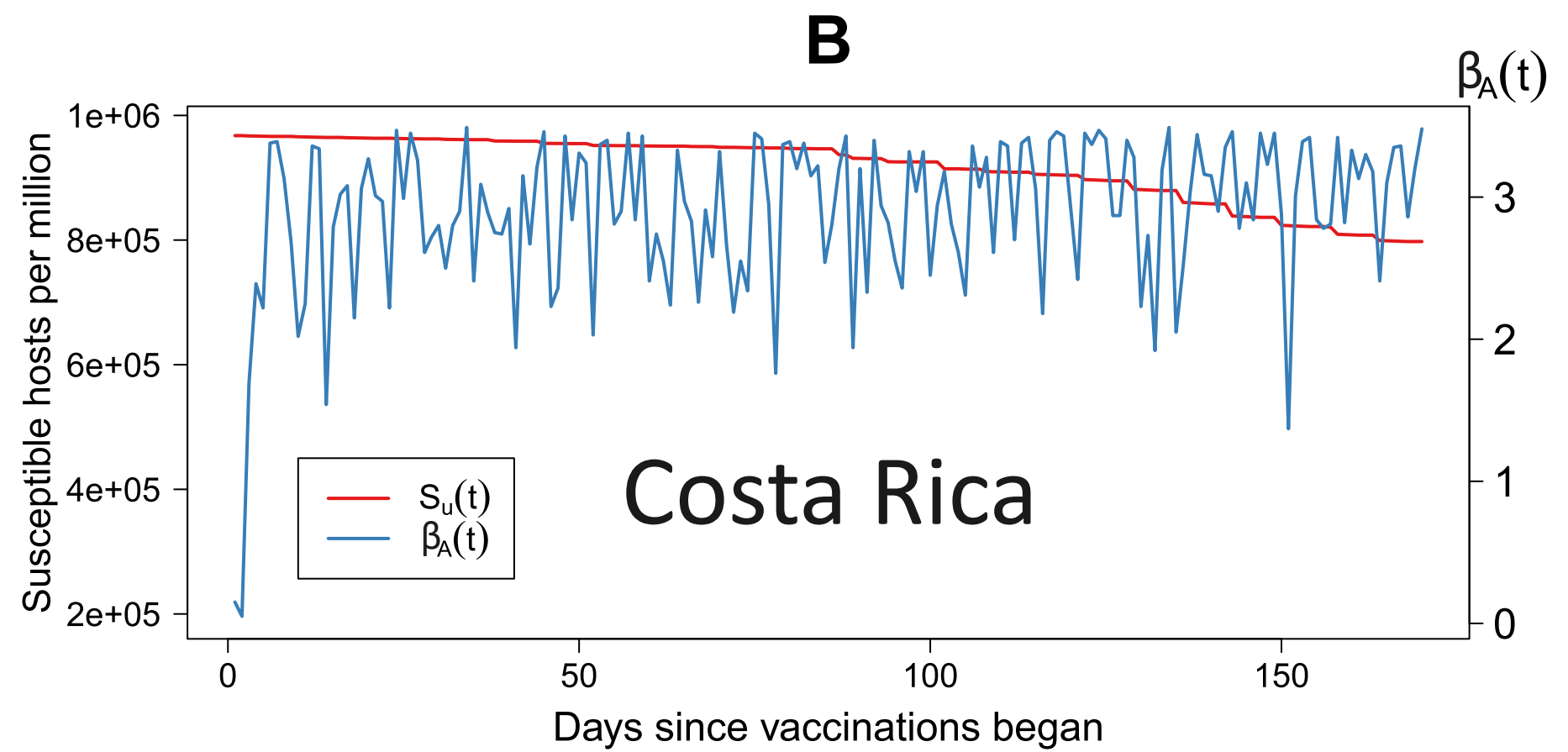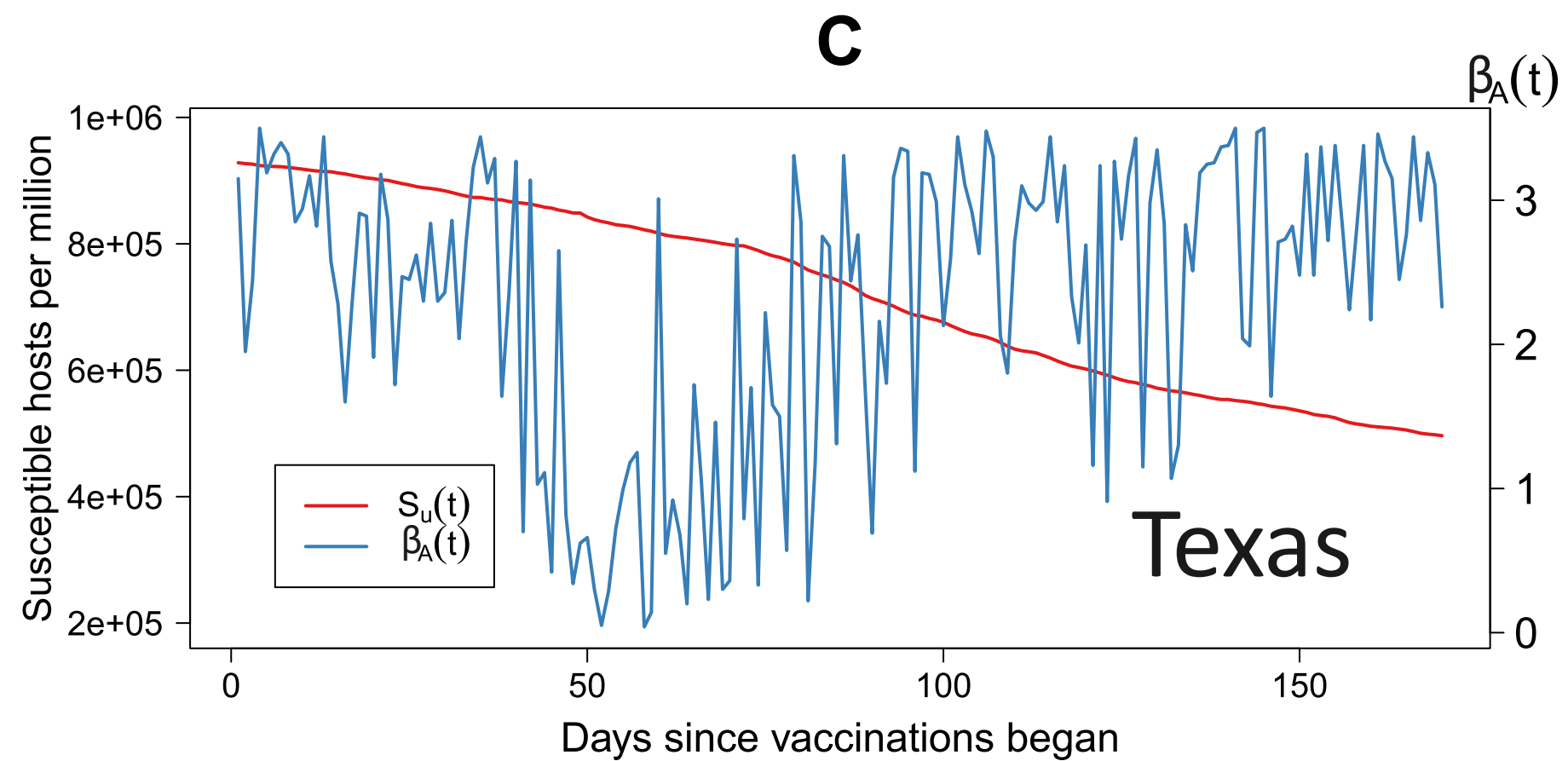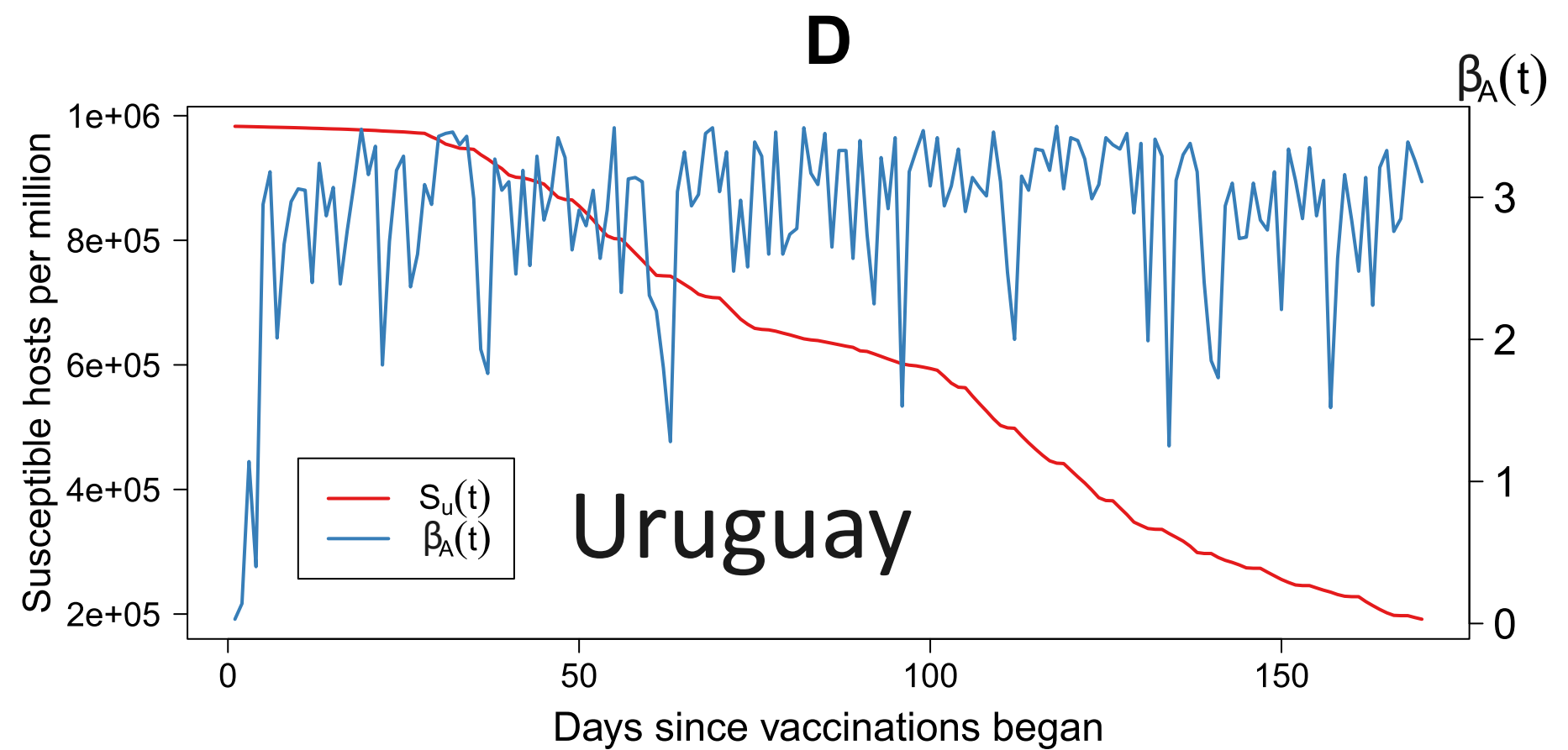

Supplement: Supplementary file 1 [file pathogens-15-00682-s001.zip › Fig_S2.pdf]

**A**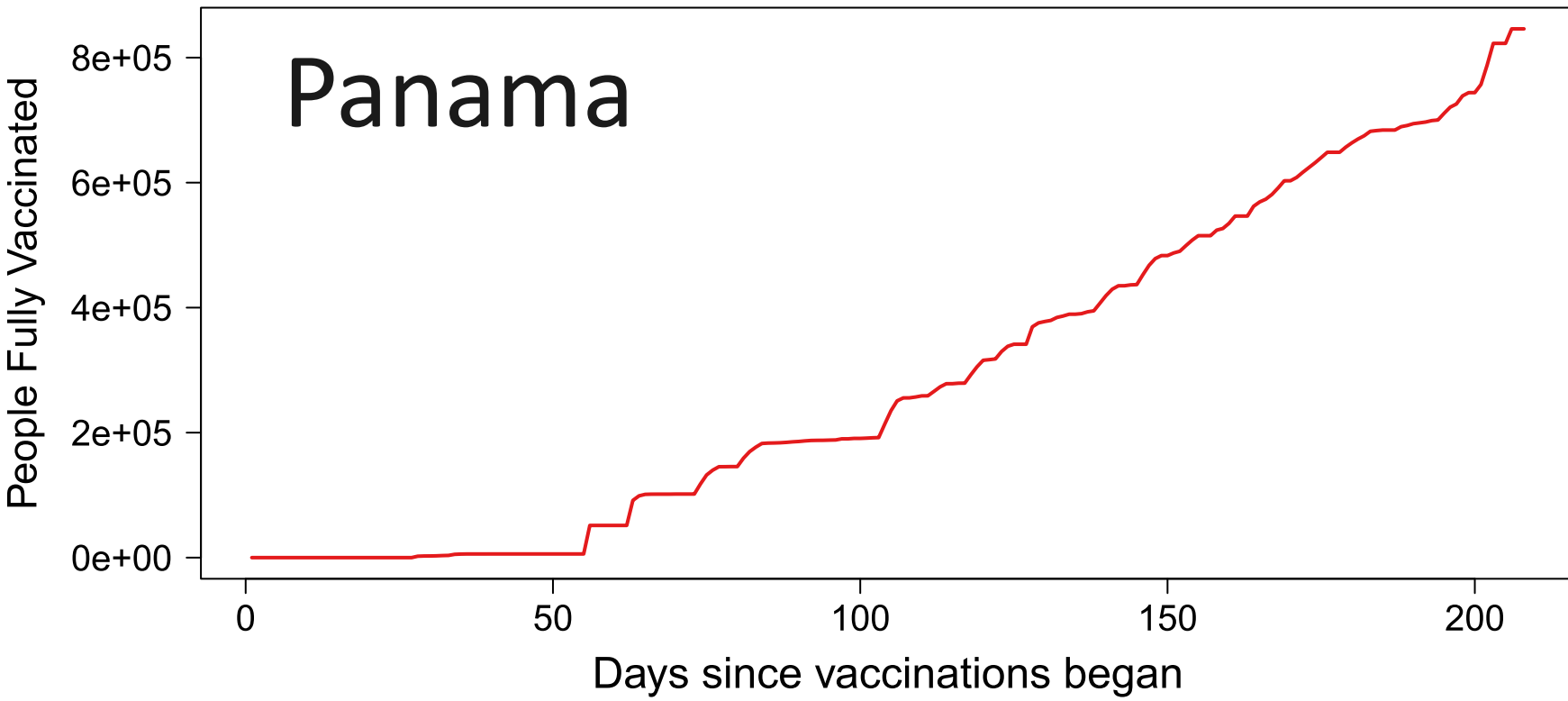**B**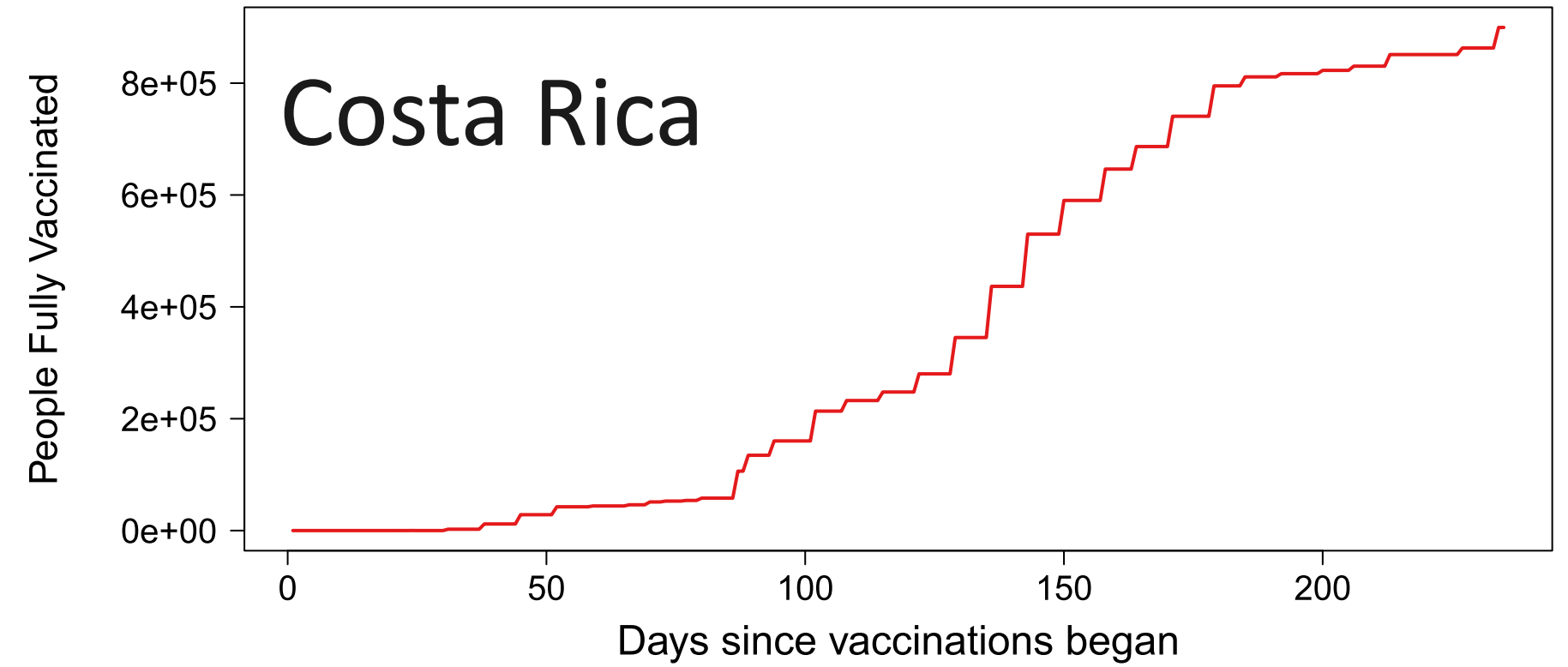**C**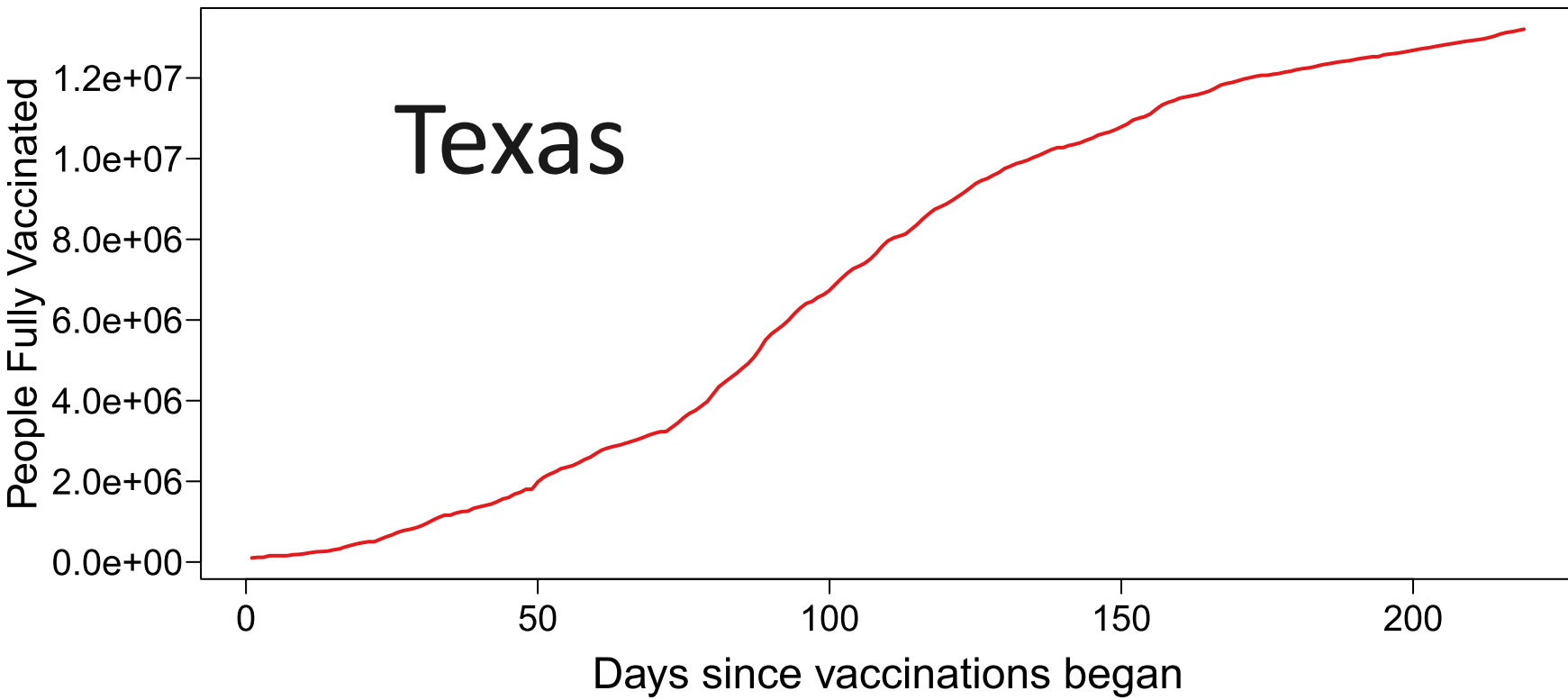**D**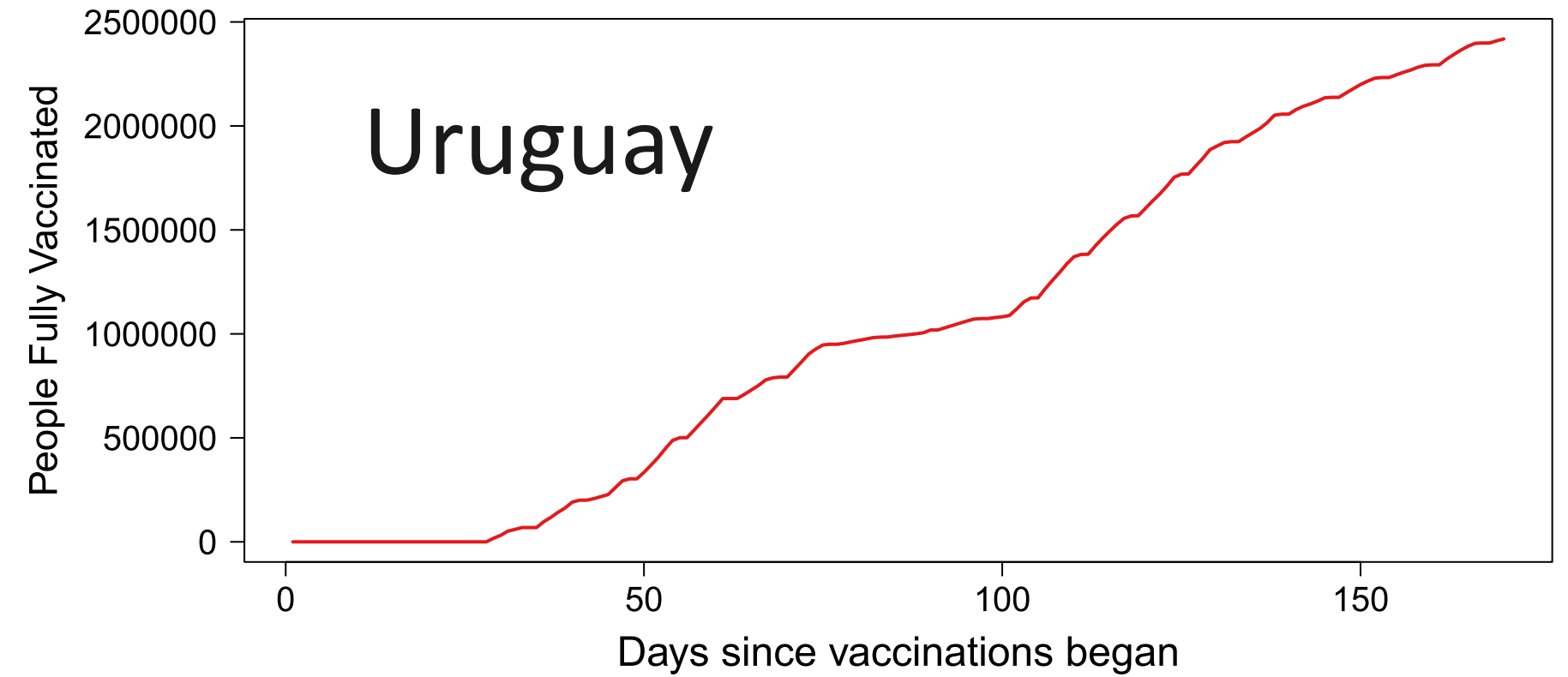

Supplement: Supplementary file 1 [file pathogens-15-00682-s001.zip › Fig_S1.pdf]
